# ArcS from *Thermococcus kodakarensis* transfers L-lysine to preQ_0_ nucleoside derivatives as minimum substrate RNAs

**DOI:** 10.1016/j.jbc.2024.107505

**Published:** 2024-06-27

**Authors:** Shu Fujita, Yuzuru Sugio, Takuya Kawamura, Ryota Yamagami, Natsuhisa Oka, Akira Hirata, Takashi Yokogawa, Hiroyuki Hori

**Affiliations:** 1 Department of Materials Science and Biotechnology, Graduate School of Science and Engineering, Ehime University, Matsuyama, Ehime, Japan; 2 Department of Chemistry and Biomolecular Science, Faculty of Engineering, Gifu University, Gifu, Gifu, Japan; 3 Institute for Glyco-core Research (iGCORE), Gifu University, Gifu, Gifu, Japan; 4 Center for One Medicine Innovative Translational Research (COMIT), Gifu University, Gifu, Gifu, Japan; 5 Department of Natural Science, Graduate School of Technology, Industrial and Social Science, Tokushima University, Tokushima, Tokushima, Japan; 6 United Graduate School of Drug Discovery and Medical Information Sciences, Gifu University, Gifu, Gifu, Japan

**Keywords:** archaeosine, RNA modification, ArcTGT, ArcS, RaSEA

## Abstract

Archaeosine (G^+^) is an archaea-specific tRNA modification synthesized *via* multiple steps. In the first step, archaeosine tRNA guanine transglucosylase (ArcTGT) exchanges the G15 base in tRNA with 7-cyano-7-deazaguanine (preQ_0_). In Euryarchaea, preQ_0_15 in tRNA is further modified by archaeosine synthase (ArcS). *Thermococcus kodakarensis* ArcS catalyzes a lysine-transfer reaction to produce preQ_0_-lysine (preQ_0_-Lys) as an intermediate. The resulting preQ_0_-Lys15 in tRNA is converted to G^+^15 by a radical S-adenosyl-L-methionine enzyme for archaeosine formation (RaSEA), which forms a complex with ArcS. Here, we focus on the substrate tRNA recognition mechanism of ArcS. Kinetic parameters of ArcS for lysine and tRNA-preQ_0_ were determined using a purified enzyme. RNA fragments containing preQ_0_ were prepared from *Saccharomyces cerevisiae* tRNA^Phe^-preQ_0_15. ArcS transferred ^14^C-labeled lysine to RNA fragments. Furthermore, ArcS transferred lysine to preQ_0_ nucleoside and preQ_0_ nucleoside 5′-monophosphate. Thus, the L-shaped structure and the sequence of tRNA are not essential for the lysine-transfer reaction by ArcS. However, the presence of D-arm structure accelerates the lysine-transfer reaction. Because ArcTGT from thermophilic archaea recognizes the common D-arm structure, we expected the combination of *T. kodakarensis* ArcTGT and ArcS and RaSEA complex would result in the formation of preQ_0_-Lys15 in all tRNAs. This hypothesis was confirmed using 46 *T. kodakarensis* tRNA transcripts and three *Haloferax volcanii* tRNA transcripts. In addition, ArcTGT did not exchange the preQ_0_-Lys15 in tRNA with guanine or preQ_0_ base, showing that formation of tRNA-preQ_0_-Lys by ArcS plays a role in preventing the reverse reaction in G^+^ biosynthesis.

To date, more than 100 modified nucleosides have been identified in tRNA (1, 2, 3). Almost all modified nucleosides in tRNA are synthesized from A, G, C, or U by chemical decorations (1, 2, 3). However, 7-deazaguanine derivatives are distinct because these modified nucleosides are initially introduced into tRNA by a base exchange reaction. Archaeosine (G^+^) contains 7-deazaguanine: G^+^ is 7-formamidino-7-deazaguanosine (2-amino-4, 7-dihydro-4-oxo-7-β-D-ribofuranosyl-*1H*-pyrro [2, 3-days] pyrimidine-5- carboximidamide) (4) (Fig. 1).

**Figure 1:**
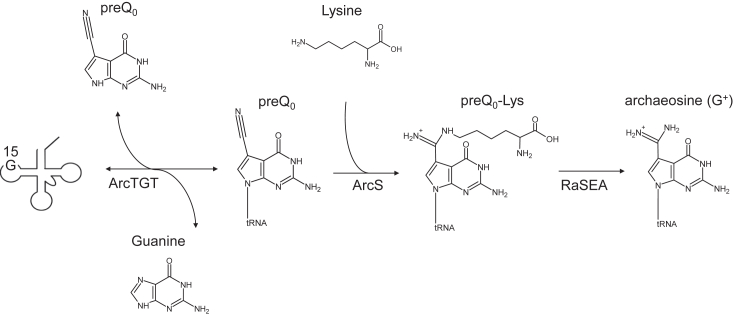
**The G**^**+**^**biosynthesis pathway of *T. kodakarensis*.** ArcTGT catalyzes the base exchange reaction from G15 to preQ_0_15 in tRNA. The resultant preQ_0_15 in tRNA is further modified to preQ_0_-Lys by ArcS. Finally, the synthesized preQ_0_-Lys in tRNA is converted to G^+^ by RaSEA.

G^+^ was first identified at position 15 in elongator tRNA^Met^ from *Thermoplasma acidophilum*, a thermo-acidophilic archaeon (5, 6), as an unknown modified nucleoside (7). It was then found in tRNAs from numerous archaea including *Haloferax volcanii* (8), *Thermoproteus neutrophilus* (9), *Sulfolobus acidocaldarius* (4, 10, 11), *Methanococcus maripuldis* (10, 11), *Pyrococcus furiosus* (10, 11), *Haloarcula marismortui* (12, 13), *Methanocaldococcus jannaschii* (14), *Sulfurisphaera tokodaii* (15), and *Thermococcus kodakarensis* (16, 17, 18, 19, 20). Furthermore, tRNA^Leu^ from *T. acidophilum* exceptionally possesses 2 G^+^ modifications at positions 13 and 15 (16, 21), while initiator tRNA^Met^ of this archaeon possesses only G^+^15 (22, 23).

A bioinformatics study predicted that G^+^15 stabilizes the L-shaped tRNA structure through reinforcement of the G15-C48 tertiary base pair (24). Consistent with this, the presence of G^+^ in the *T. kodakarensis* tRNA^Gln^ transcript increases the melting temperature in the presence of 100 μM and 10 mM MgCl_2_ (20). Furthermore, i*n T. kodakarensis*, a deletion strain of the *arcTGT* gene, which encodes archaeosine tRNA guanine transglucosylase (ArcTGT), an enzyme for the first step of G^+^ synthesis in tRNA cannot grow at high temperatures (17). These studies reveal that the G^+^15 modification in tRNA stabilizes the L-shaped tRNA structure and is required for the survival of thermophilic archaea at high temperatures.

The biosynthetic pathway of G^+^ in tRNA consists of multiple steps (Fig. 1). In the first step, ArcTGT exchanges the G15 base in tRNA with 7-cyano-7-deazaguanine (preQ_0_) (25, 26). ArcTGT proteins and their genes have been experimentally identified in several archaea including *H. volcanii* (25, 26), *M*. *janaschii* (27), *Pyrococcus horikoshii* (28, 29, 30), *P*. *furiosus* (31), *Methanosarcina barkeri* (31), *Methanosarcina acetivorans* (32, 33), *T. acidophilum* (16), and *T. kodakarensis* (16), which is consistent with the wide spread of G^+^ in archaeal tRNAs. The enzymatic properties and tRNA recognition mechanisms of ArcTGT from several archaea have been reported (16, 25, 26, 27, 28, 31, 32, 33). Furthermore, crystal structure studies of ArcTGT and the complex between ArcTGT and tRNA have been performed (29, 30).

The second and subsequent steps of the G^+^ biosynthesis pathway differ between archaea species. In Euryarchaea, preQ_0_15 in tRNA is further modified by archaeosine synthase (ArcS) (Fig. 1). ArcS was initially found by amino acid sequence homology with ArcTGT (34) and then analysis of tRNAs from a *H. volcanii arcS* gene deletion strain revealed that ArcS is involved in G^+^ biosynthesis (34). ArcS from *M*. *janaschii* uses NH_4_^+^, asparagine, or glutamine as a nitrogen source and directly synthesizes G^+^15 from preQ_0_ in tRNA (34). In contrast, ArcS proteins from *T. acidophilum*, *M. acetivorans*,and *T. kodakarensis* catalyze a lysine-transfer reaction and synthesize preQ_0_-lysine (preQ_0_-Lys) at position 15 in tRNA as an intermediate in G^+^ biosynthesis (19) (Fig. 1). ArcS proteins from *T. acidophilum*, *M. acetivorans*,and *T. kodakarensis* do not use NH_4_^+^, asparagine, or glutamine as a nitrogen source (19). The resulting preQ_0_-Lys15 in tRNA is converted to G^+^15 by a radical S-adenosyl-L-methionine enzyme for archaeosine formation (RaSEA) (19). Because 196 Euryarchaea species including *M*. *janaschii* possess the gene set of *arcS* and *RaSEA*, the latter pathway (combination of ArcS and RaSEA) may be mainly used in the G^+^15 formation in living cells (19). In contrast, most Crenarchaea do not possess the *arcS* gene (35). In the case of *Pyrobaculum calidifontis* (a Crenarchaea), a QueF-like protein synthesizes G^+^15 in tRNA from preQ_0_15 using NH_4_^+^ as a nitrogen donor (36, 37).

These progresses described above in the study of G^+^ formation in tRNA have been achieved over the last 3 decades. However, the mechanism of substrate tRNA recognition by ArcS is still unknown. To address this issue, we have performed biochemical analyses which are described in this study.

## Results

### Purification of recombinant ArcTGT, ArcS, and RaSEA complex

To measure the enzymatic activity of ArcS, tRNA containing preQ_0_ at position 15 (tRNA-preQ_0_15), which is prepared using ArcTGT, is required as a substrate. However, ArcTGT from *T. acidophilum* is not expressed as a soluble protein in *Escherichia coli* cells (16). Furthermore, because ArcTGT from *M. acetivorans* is a split-type (32), the expression of two protein subunits in *E. coli* cells is required. The lysine-transfer activity of ArcS from *T. kodakarensis* was confirmed in our previous report (19). In contrast, ArcS from *M. janaschii* uses NH_4_^+^, asparagine, or glutamine as a nitrogen source (34). Therefore, we used the set of ArcTGT, and ArcS and RaSEA complex from *T. kodakarensis* in this study. ArcS from *T. kodakarensis* forms a complex with RaSEA (19). Although recombinant ArcS protein expressed in *E. coli* cells is soluble in the absence of RaSEA, this free ArcS protein does not catalyze the lysine-transfer reaction (19). Only the complex of ArcS and RaSEA demonstrates the lysine-transfer activity (19). Furthermore, ArcTGT does not form a complex with ArcS. To show this, we performed a co-expression experiment in *E. coli* (Fig. S1). Three proteins (ArcTGT, His x 6-ArcS, and RaSEA) were co-expressed in *E. coli* cells, co-purified by NiNTA Superflow column chromatography, and analyzed by 10% SDS-PAGE. RaSEA was co-purified with His x 6-ArcS, showing that ArcS and RaSEA form a complex. In contrast, ArcTGT was not purified with His x 6-ArcS. Thus, this experimental result shows that ArcS does not form a complex with ArcTGT. *T. kodakarensis* ArcTGT and ArcS and RaSEA complex were expressed in *E. coli* cells and purified as shown in Fig. S2, *A* and *B*. It should be mentioned that these enzymes were purified under aerobic conditions. Thus, RaSEA is in an apo form, which does not contain a Fe-S cluster. Therefore, preQ_0_-Lys15 in tRNA is not converted to G^+^15 in the reaction mixture containing ArcS and RaSEA complex, and formation of preQ_0_-Lys15 in tRNA by ArcS can be directly monitored.

### Measurement of kinetic parameters of lysine transfer reaction

**Figure 2:**
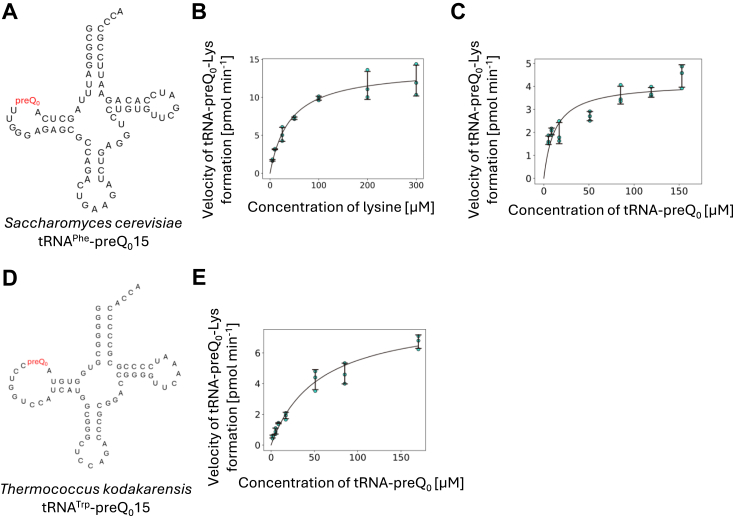
**Measurement of kinetic parameters of lysine-transfer reaction of ArcS.***A*, cloverleaf representation of *S*. *cerevisiae* tRNA^Phe^-preQ_0_15. The position of preQ_0_ is highlighted in *red*. *B*, kinetic parameters for lysine were measured at 60 °C using tRNA^Phe^-preQ_0_15 and ^14^C-labeled lysine. *C*, kinetic parameters for *S*. *cerevisiae* tRNA^Phe^-preQ_0_15 were measured at 60 °C. *D*, cloverleaf representation of *T. kodakarensis* tRNA^Trp^-preQ_0_15. The position of preQ_0_ is highlighted in *red*. *E*, kinetic parameters for *T. kodakarensis* tRNA^Trp^-preQ_0_15 were measured at 60 °C.

ArcS was originally found *via* its amino acid sequence homology with ArcTGT (34). Initially, therefore, we expected that the substrate tRNA recognition mechanism of ArcS might resemble that of ArcTGT. Fortunately, the substrate tRNA recognition mechanism of ArcTGT from a thermophilic archaeon (*P. horikoshii*) has been reported (28). ArcTGT from *P. horikoshii* does not recognize the L-shaped tRNA structure and nucleotide sequences (except for G15) in the D-loop but rather recognizes the ribose-phosphate backbone of the D-arm (28). However, the three-dimensional structure of tRNA from *T. kodakarensis* has not been reported. Therefore, we selected *Saccharomyces cerevisiae* tRNA^Phe^-preQ_0_15 (Fig. 2*A*) as a model substrate because the three-dimensional structure of this tRNA is well established (38). It should be mentioned that native tRNA^Phe^ from *S. cerevisiae* does not possess the G^+^15 modification (38). The substrate tRNA^Phe^ transcript, in which preQ_0_ was present at position 15, was prepared using the base exchange reaction of ArcTGT. We measured the kinetic parameters of ArcS for lysine (Fig. 2*B*) and tRNA^Phe^-preQ_0_15 (Fig. 2*C*). The values of kinetic parameters are given in Table 1. The Km value of ArcS for lysine was determined to be 40.9 μM (Fig. 2*B*), which is comparable to that of *E. coli* and *S. cerevisiae* lysyl-tRNA synthetases for lysine (27 μM and 38 μM, respectively; ref. (39, 40)). Although the concentration of lysine in *T. kodakarensis* cells is unknown, this Km value of ArcS is normal for a lysine-related enzyme. In contrast, the Km value of ArcS for tRNA^Phe^-preQ_0_15 was determined to be 12.3± 3.2 μM (Fig. 2*C* and Table 1). This value is considerably large for the Km value for substrate tRNA of a tRNA modification enzyme. For example, the Km value of *P. horikoshii* ArcTGT for tRNA^Val^ transcript has been reported to be 0.57 μM (28). We suspected that the large Km value for tRNA^Phe^-preQ_0_ might be caused by the sequence of *S. cerevisiae* tRNA^Phe^. As described above, *S. cerevisiae* tRNA^Phe^-preQ_0_ is an artificial substrate for ArcS. To address this issue, we prepared *T. kodakarensis* tRNA^Trp^-preQ_0_ transcript and performed the kinetic studies using this tRNA transcript (Fig. 2*D*). In *T. kodakarensis* tRNAs, the presence of G^+^15 has only been confirmed in tRNA^Trp^ (18). As shown in Figure 2*E* and Table 1, the Km value for *T. kodakarensis* tRNA^Trp^-preQ_0_ transcript was determined to be 54.9 μM, which is comparable to that for *S. cerevisiae* tRNA^Phe^-preQ_0_ transcript. Thus, the large Km values of ArcS are common to both artificial and natural substrate tRNAs. However, the Kcat values of ArcS for *S. cerevisiae* tRNA^Phe^-preQ_0_ and *T. kodakarensis* tRNA^Trp^-preQ_0_ were determined to be around 3.7 min^−1^ and 7.5 min^−1^, respectively. These Kcat values for lysine-transfer reaction by ArcS are larger than that of tRNA-preQ_0_15 formation by ArcTGT: the Kcat of *P. horikoshii* ArcTGT is calculated to be 4.9 min^−1^ from the kinetic values reported (28). Therefore, the velocity of the lysine-transfer reaction mediated by ArcS may be sufficient for tRNA-preQ_0_-Lys15 formation in living cells.

**Table 1:** Kinetic parameters for lysine and substrate RNAs

Substrate	Km [μmol]	Kcat [min^−1^]	Relative Kcat/Km
L-lysine	40.9 ± 8.7	6.1 ± 0.7	
*S. cerevisiae* tRNA^Phe^-preQ_0_	12.3 ± 3.2	3.7 ± 0.4	1.00
*T. kodakarensis* tRNA^Trp^-preQ_0_	54.9 ± 23.4	7.5 ± 0.9	0.45
21 nt RNA-preQ_0_ fragment	18.4 ± 7.4	14.8 ± 1.8	2.67
64 nt RNA-preQ_0_ fragment	28.3 ± 6.2	27.1 ± 1.9	3.18
5′ P-preQ_0_ nucleotide	433.4 ± 6.0	23.2 ± 3.7	0.18

Relative Kcat/Km value for *S. cerevisiae* tRNA^Phe^ is expressed as 1.00.

### ArcS does not require the L-shaped tRNA structure for the lysine-transfer reaction

**Figure 3:**
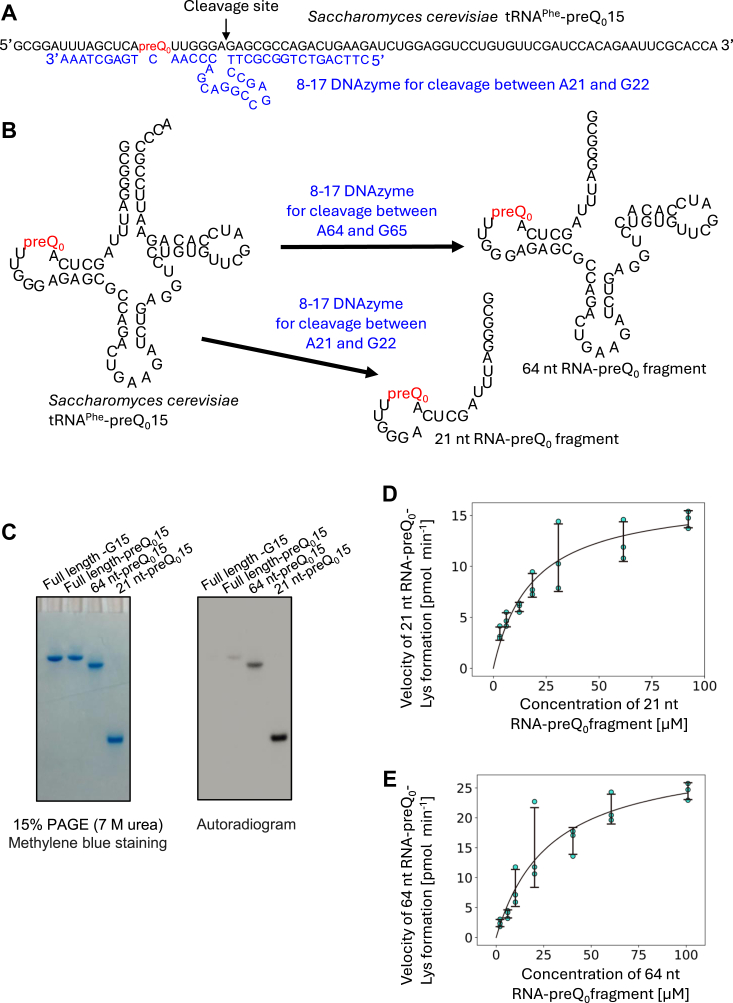
**ArcS does not require the L-shaped tRNA structure for the lysine-transfer reaction.***A*, RNA fragments, in which preQ_0_ was present, were prepared from tRNA^Phe^-preQ_0_15 by 8-17 DNAzymes. The sequence of the 8-17 DNAzyme for cleavage between A21 and G22 is shown in *blue*. The position of preQ_0_ is highlighted in red. This DNAzyme cleaves the phosphodiester bond between A21 and G22. *B*, in this study, two 8-17 DNAzymes were used. One DNAzyme cleaves the phosphodiester bond between A21 and G22 and the other cleaves that between A64 and G65. The resultant RNA fragments were purified using 10% PAGE (7 M urea). *C*, ^14^C-lysine incorporation into RNA fragments was assayed. Full-length tRNA^Phe^-G15 and -preQ_0_15 were used as negative and positive controls, respectively. Kinetic parameters for 21 nt (*D*) and 64 nt (*E*) RNA fragments were measured.

To clarify the recognition site(s) of ArcS in tRNA, we prepared RNA fragments derived from tRNA^Phe^-preQ_0_15 by deoxyribozyme (DNAzyme) reaction (41) (Fig. 3*A*). We selected 8-17 DNAzyme (42) for this study because it is able to cleave AG sequences in RNAs site-specifically (42, 43). We designed two 8-17 DNAzymes and cleaved tRNA^Phe^-preQ_0_15: one cleaves between A21 and G22 and the other cleaves between A64 and G65 (Fig. 3*B*). The resultant preQ_0_-RNA fragments (21 nt and 64 nt) were purified by 10% polyacrylamide gel containing 7 M urea electrophoresis [10% PAGE (7 M urea)]. We used full-length tRNA^Phe^-G15 and tRNA^Phe^-preQ_0_15 transcripts as negative and positive controls, respectively. These RNAs (8.5 μM each) were incubated with 0.11 μM ArcS-RaSEA complex and 100 μM ^14^C-labeled lysine at 60 °C for 10 min. The RNAs were treated with phenol-chloroform, recovered by ethanol precipitation, and then 0.03 A260 units of RNAs were analyzed by 10% PAGE (7 M urea) (Fig. 3*C* left). The autoradiogram of the same gel was collected (Fig. 3*C* right). Under the condition tested, the band intensities show relative velocities of ^14^C-lysine-transfer to RNAs. As shown in Figure 3*C* right, ^14^C-lysine was transferred to all RNAs containing preQ_0_. In contrast, ^14^C-lysine was not transferred to full-length tRNA^Phe^-G15 (negative control). These results clearly show that ArcS does not require the L-shaped tRNA structure for the lysine-transfer reaction. Furthermore, to our surprise, the band intensities in the autoradiogram showed that the incorporation of ^14^C-lysine into the 21 nt and 64 nt RNA fragments was more efficient than that into the full-length tRNA-preQ_0_15. To confirm this, we performed kinetic studies using the 21 nt and 64 nt RNA fragments (Fig. 3, *D* and *E*, and Table 1). As shown in Figure 3*D* and Table 1, both the Km and Kcat values for the 21 nt RNA fragment were improved relative to those for full-length tRNA^Phe^-preQ_0_15. Thus, this analysis reveals that the L-shaped tRNA structure has a negative effect on the lysine-transfer reaction of ArcS. Furthermore, in the 21 nt RNA fragment, the D-arm structure as well as the L-shaped tRNA structure is disrupted. Therefore, it is also clear that the D-arm structure is not essential for the lysine-transfer reaction by ArcS. However, the Km and Kcat values for the 64 nt RNA fragment revealed that the 64 nt RNA fragment was the best substrate of the RNAs tested (Table 1). In the 64 nt RNA fragment, the D-arm structure is not disrupted. These results show that the presence of a D-arm structure accelerates the velocity of the lysine-transfer reaction by ArcS although the D-arm structure is not essential for the lysine-transfer reaction.

### ArcS can transfer lysine to preQ_0_ nucleoside and the presence of 5′-phosphate accelerates the reaction

**Figure 4:**
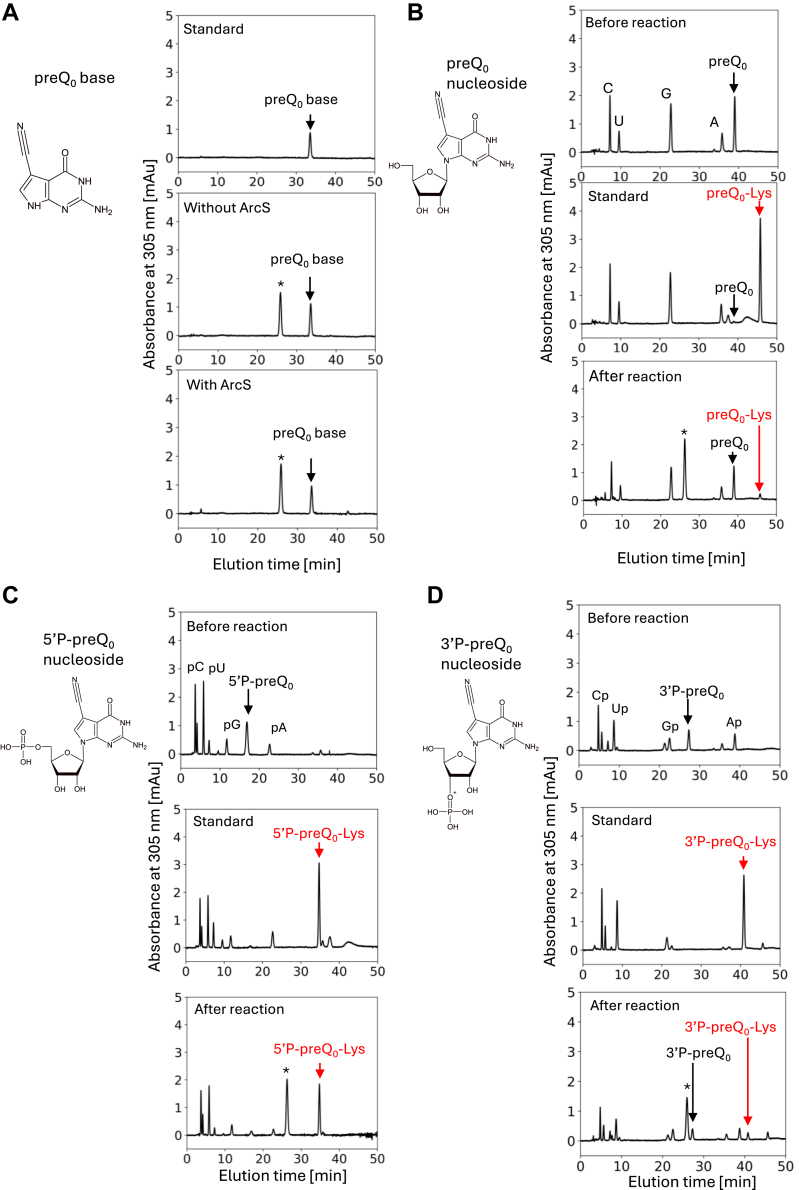
**preQ**_**0**_**nucleoside and its derivatives are the substrates of ArcS.** The structures of preQ_0_ and its derivatives are illustrated in each panel. Asterisks mark a derivative from 2-mercaptoethanol. This derivative is non-enzymatically synthesized during incubation at 60 °C for 2 h. *A*, ArcS does not transfer lysine to preQ_0_ base. *B*, ArcS transfers lysine to preQ_0_ nucleoside very slowly. *C*, the presence of 5′-phosphate in 5′P-preQ_0_ nucleoside accelerates the lysine-transfer reaction of ArcS. *D*, the presence of 3′-phosphate in 3′P-preQ_0_ nucleoside does not accelerate the lysine-transfer reaction of ArcS.

ArcS can transfer lysine to a 21 nt RNA fragment containing preQ_0_. This result prompted us to investigate the identity of the minimum substrate RNA for ArcS (Fig. 4). Because preQ_0_ and its derivatives absorb ultra-violet light at 305 nm (UV_305nm_), the formation of lysine-adducts can be monitored by absorbance of UV_305nm_ using high-performance liquid column (HPLC) chromatography. The elution time of preQ_0_ and its derivatives were determined using standard compounds, which were prepared by organic chemistry or nuclease digestion of full-length tRNA^Phe^-preQ_0_-Lys (details are described in Experimental Procedures). When preQ_0_ base was used as a substrate, ArcS did not transfer lysine to preQ_0_ base (Fig. 4*A* lower). It should be mentioned that the peak at 25.8 min (marked by an asterisk in Fig, 4) is a result of the presence of 2-mercaptoethanol in the reaction mixture. Therefore, this peak was also observed in the negative control, to which ArcS was not added (Fig. 4*A* middle). When 34.0 μM preQ_0_ nucleoside was incubated with 0.23 μM ArcS and 200 μM lysine at 60 °C for 2 h, a small peak of preQ_0_-Lys nucleoside appeared (Fig. 4*B* lower). The standard marker (Fig. 4*B* middle) was prepared by nuclease P1 and bacterial alkaline phosphatase double digestions of tRNA^Phe^-preQ_0_-Lys. Thus, the minimum substrate for ArcS is preQ_0_ nucleoside. Furthermore, when phosphate was attached to the 5′-OH of preQ_0_ nucleoside (*i. e*. preQ_0_ nucleoside 5′-monophosphate; 5′P-preQ_0_), a clear peak of preQ_0_-Lys nucleoside 5′-monophosphate (5′P-preQ_0_-Lys) appeared (Fig. 4*C* lower). When preQ_0_ nucleoside was used as a substrate, a very small preQ_0_-Lys peak appeared after 2 h incubation (Fig. 4*B*). In contrast, when 5′P-preQ_0_ nucleoside was used, almost all 5′P-preQ_0_ nucleoside was converted to 5′P-preQ_0_-Lys (Fig. 4*C*). These data show that the presence of 5′-phosphate is required for the efficient lysine-transfer reaction. Furthermore, when phosphate was attached to the 3′-OH of preQ_0_ nucleoside (preQ_0_ nucleoside 3′-monophosphate; 3′P-preQ_0_), the formation of lysine-adduct (3′P-preQ_0_-Lys) by ArcS was not increased (Fig. 4*D*). Thus, 3′-phosphate is not involved in the substrate recognition by ArcS. Taking these experimental results together, we conclude that the minimum substrate for ArcS is preQ_0_ nucleoside and that the presence of 5′-phosphate is required for the efficient lysine-transfer reaction of ArcS.

### Kinetic study of the lysine-transfer reaction to 5′P-preQ_0_-Lys mediated by ArcS

**Figure 5:**
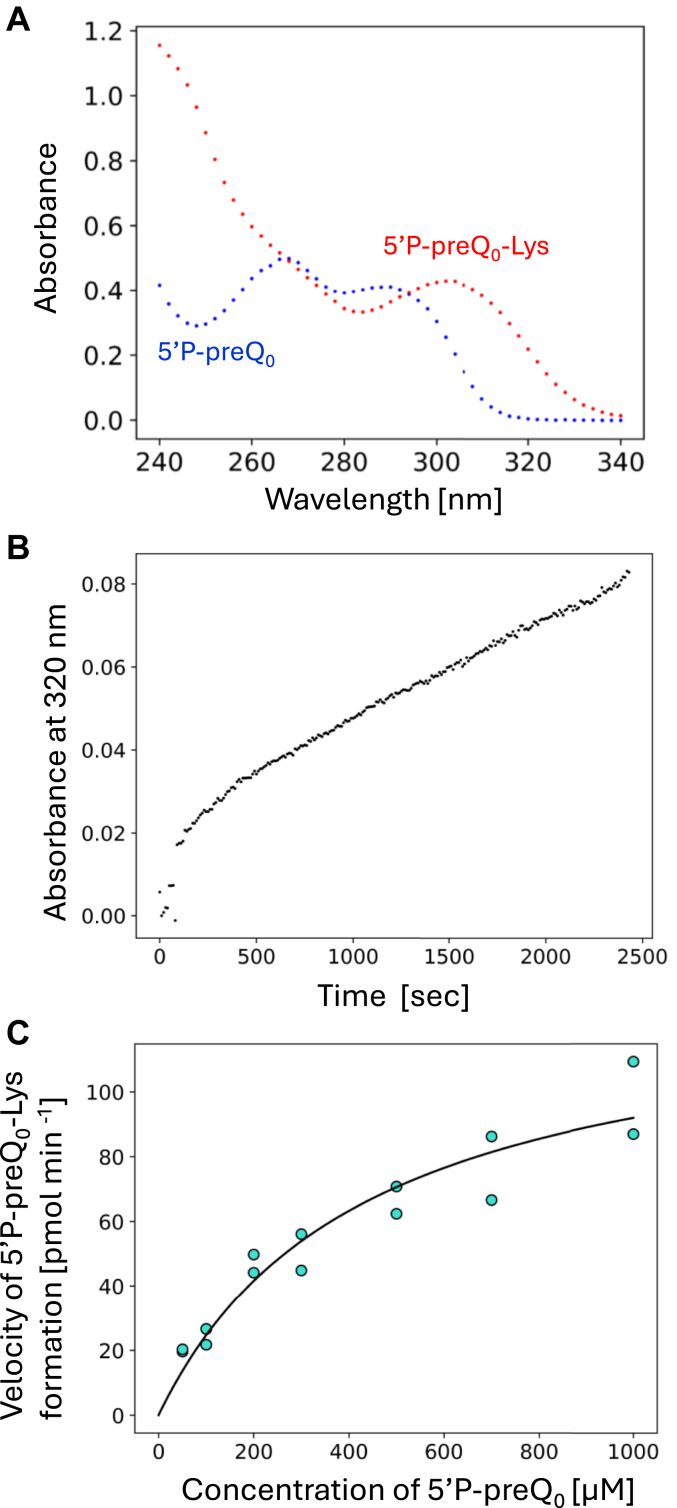
**Measurement of kinetic parameters of ArcS for 5′P-preQ**_**0**_**.***A*, UV-absorption spectra of 5′P-preQ_0_-Lys (*red*) and 5′P-preQ_0_ (*blue*) at 60 °C. *B*, the initial velocity of formation of 5′P-preQ_0_-Lys was monitored by absorbance at 320 nm. *C*, kinetic parameters of ArcS for 5′P-preQ0 nucleoside were determined spectroscopically.

Because the substrate is a nucleotide (or nucleoside), measurement of the velocity of lysine-adduct formation by conventional filter assay with ^14^C-lysine is difficult. Therefore, we tested whether the kinetic parameters of ArcS for 5′P-preQ_0_ could be measured spectroscopically. Initially, we measured the ultra-violet light absorption spectra of 5′P-preQ_0_ (Fig. 5*A* blue) and 5′P-preQ_0_-Lys (Fig. 5*A* red): these compounds were prepared by organic synthesis. As shown in Figure 5*A*, 5′ P-preQ_0_ did not absorb UV_320 nm_. In contrast, 5′P-preQ_0_-Lys absorbs UV_320nm_. Therefore, we hypothesized that the velocity of 5′P-preQ_0_-Lys formation could be measured by an increase in absorbance at 320 nm. To verify this idea, we mixed 0.11 μM ArcS-RaSEA complex, 50 μM 5′ P-preQ_0_ and 200 μM lysine and then monitored the change of absorbance at 320 nm at 60 °C (Fig. 5*B*). It should be mentioned that 2-mercaptoethanol was not added into the reaction mixture because 2-mercaptoethanol is converted to a derivative, which absorbs UV_320 nm_ (this derivative is marked by asterisks in Fig. 4). As shown in Figure 5*B*, the initial velocity of 5′ preQ_0_-Lys formation could be measured. We determined the kinetic parameters of ArcS for 5′P-preQ_0_ using this method (Fig. 5*C* and Table 1). The Km value for 5′P-preQ_0_ was very large (433.4 μM). However, the Kcat value for 5′P-preQ_0_ was comparable to that for a 21 nt or 64 nt RNA fragment. Because the Kcat values for 5′P-preQ_0_ and RNA fragments are higher than that for full-length tRNA transcript (Table 1), we propose that the L-shaped tRNA structure may preturb the lysine-transfer reaction mediated by ArcS.

### The combination of ArcTGT and ArcS and RaSEA complex can form preQ_0_-Lys in all tRNA transcripts from *T. kodakarensis*

**Figure 6:**
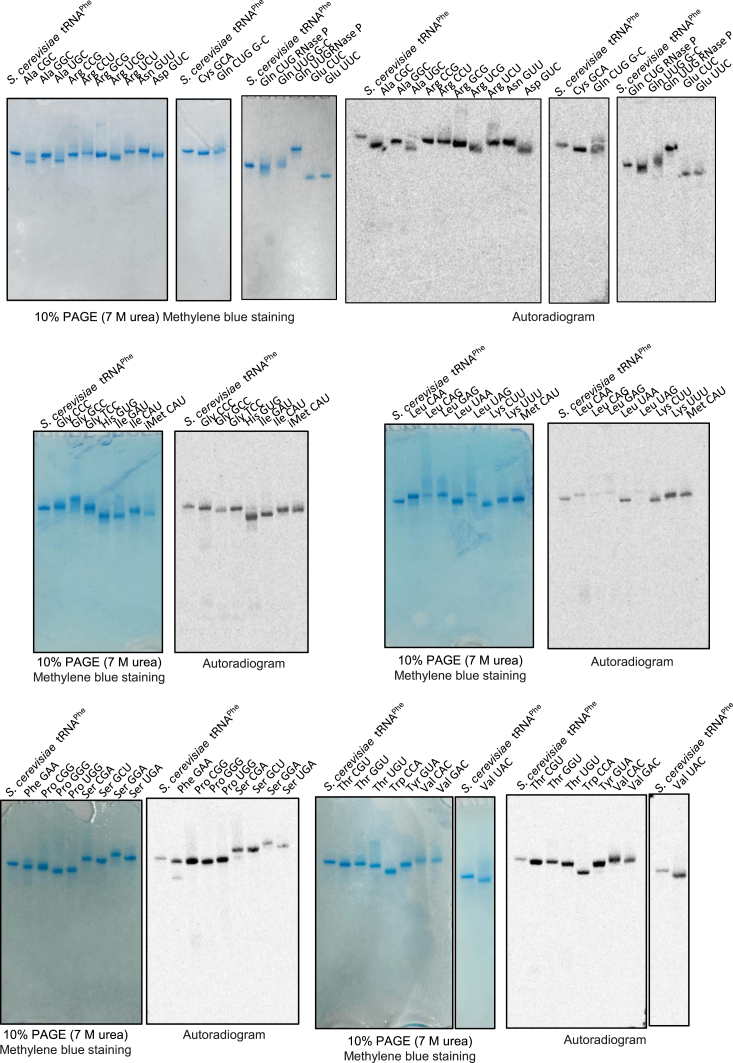
**Combination of ArcTGT and ArcS and RaSEA complex from *T. kodakarensis* transfers**^**14**^**C-labeled lysine to all tRNA transcripts tested.** In the *T. kodakarensis* genome, 46 tRNA genes are present. 46 tRNA transcripts were prepared and then tested to determine whether a combination of ArcTGT and ArcS and RaSEA complex transferred ^14^C-labeled lysine into them. After the reaction, 0.01 A260 units of tRNAs were loaded onto the gels. The RNAs were visualized by methylene blue staining and autoradiograms of the gels were obtained. *Saccharomyces cerevisiae* tRNA^Phe^ was used as a control in each gel. Abbreviations are as follows: iMet, initiator tRNA^Met^ transcript; Met, elongator tRNA^Met^ transcript.

**Figure 7:**
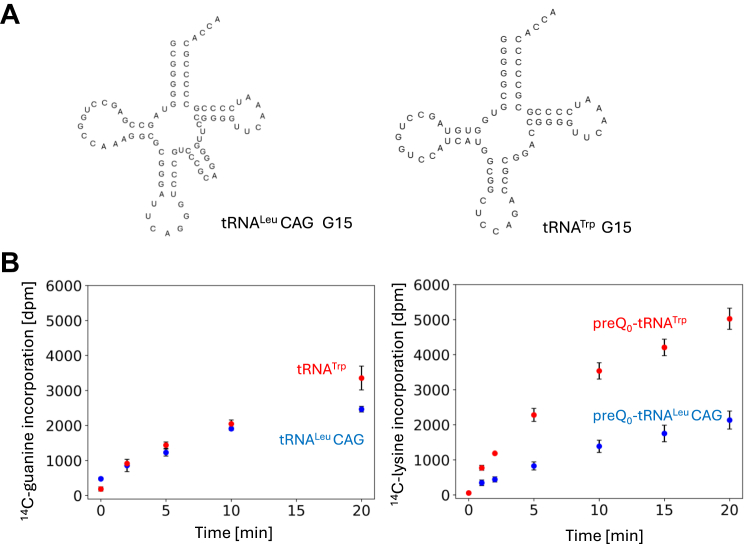
**The slow preQ**_**0**_**-Lys formation in tRNA**^**Leu**^**CAG is caused by slow lysine-transfer of ArcS.***A*, cloverleaf structures of tRNA^Leu^CAG and tRNA^Trp^ transcripts are shown. *B*, ^14^C-guanine incorporations into tRNA^Leu^CAG (*blue*) and tRNA^Trp^ (*red*) transcripts by ArcTGT are compared (*left*). ^14^C-lysine incorporations into tRNA^Leu^CAG-preQ_0_ (*blue*) and tRNA^Trp^-preQ_0_ (*red*) transcripts by ArcS are compared. The data were obtained from three independent experiments. Error bars show the standard deviations.

ArcS can act on preQ_0_ nucleoside. This result suggests that the tRNA specificity of preQ_0_-Lys15 formation by ArcS may be determined by the substrate tRNA specificity of ArcTGT. In other words, if ArcTGT introduces a preQ_0_ base into tRNA, the preQ_0_ may be modified to preQ_0_-Lys by ArcS irrespective of the sequences of the tRNAs. As described in the previous section, ArcTGT from *P. horikoshii* does not recognize the L-shaped tRNA structure and nucleotide sequences (except for G15) in the D-loop but rather recognizes the ribose-phosphate backbone of the D-arm (28). If *T. kodakarensis* ArcTGT possesses the same tRNA specificity, the combination of ArcTGT and ArcS and RaSEA complex would modify all tRNA transcripts from *T. kodakarensis*. In the *T. kodakarensis* genome, 46 tRNA genes are encoded (44). To confirm the above idea, we prepared 46 tRNA transcripts using T7 RNA polymerase (45) and tested whether ArcTGT and ArcS and RaSEA complex form preQ_0_-Lys in these tRNA transcripts (Figs. 6 and S3). In this experiment, *S. cerevisiae* tRNA^Phe^ transcript was used as a control. As shown in Fig. 6, ^14^C-lysine was incorporated into all tRNA transcripts tested. Thus, *T. kodakarensis* ArcTGT and ArcS and RaSEA complex act on all tRNA species *in vitro*. However, the velocities of preQ_0_-Lys formation differed according to the tRNA species. For example, preQ_0_-Lys formation into tRNA^Leu^CAG transcript was clearly slower than that into other tRNA transcripts (Fig. 6). In all tRNA species from *T. kodakarensis*, the presence of G^+^15 has only been confirmed in tRNA^Trp^ (18). Therefore, we prepared tRNA^Leu^CAG and tRNA^Trp^ transcripts (Fig. 7*A*) and tested the ^14^C-guanine exchange reactions of ArcTGT (Fig. 7*B* left). There was no significant difference between the velocities of the ^14^C-guanine exchange reaction for these tRNA transcripts (Fig. 7*B* left). In contrast, the velocity of ^14^C-lysine-transfer to preQ_0_-tRNA^Trp^ mediated by ArcS (red) was faster than that to preQ_0_-tRNA^Leu^CAG (blue) (Fig. 7*B* right). Thus, the slow preQ_0_-Lys formation for tRNA^Leu^CAG transcript is mainly caused by a slow lysine-transfer by ArcS. This result shows that velocities of lysine-transfer by ArcS differ according to tRNA species although ArcS can act on preQ_0_ nucleoside. Moreover, ArcTGT changes the structure of substrate tRNA from the L-shaped structure to the so-called the λ-form (30). Therefore, when a combination of ArcTGT and ArcS and RaSEA complex was used (Fig. 6), the λ-form structure formed by ArcTGT might affect the formation of preQ_0_-Lys by the ArcS and RaSEA complex.

### *T. kodakarensis* ArcS can transfer lysine to mesophilic archaeal tRNAs, which do not possess the G^+^15 modification in cells

**Figure 8:**
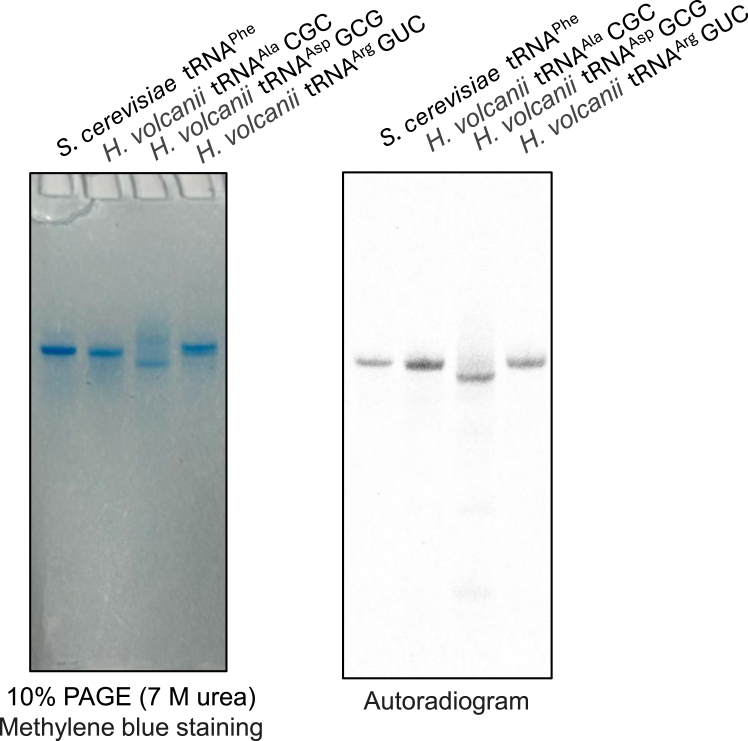
**Combination of ArcTGT and ArcS and RaSEA complex from *T. kodakarensis* transfers**^**14**^**C-labeled lysine to *H. volcanii* tRNA transcripts, which do not possess G**^**+**^**modification in living cells.** Native *H. volcanii* tRNA^Ala^, tRNA^Asp^ and tRNA^Arg^ do not possess the G^+^15 modification. The combination of ARcTGT and ArcS and RaSEA complex from *T. kodakarensis* transfers ^14^C-lysine to these tRNA transcripts. *Saccharomyces cerevisiae* tRNA^Phe^ was used as a positive control. The RNAs were visualized by methylene blue staining (*left*) and an autoradiogram of the gel was obtained (*right*).

Several tRNAs from mesophilic archaea do not possess the G^+^15 modification. For example, in the case of *H. volcanii*, tRNA^Asp^GUC, tRNA^Ala^CGC and tRNA^Arg^GCG possess unmodified G15 (8). Because the set of *T. kodakarensis* ArcTGT and ArcS and RaSEA complex acts on all tRNA species, we tested whether this set forms preQ_0_-Lys in *H. volcanii* tRNA^Asp^GUC, tRNA^Ala^CGC and tRNA^Arg^GCG transcripts. As shown in Figure 8, ^14^C-lysine was transferred to *H. volcanii* tRNA^Asp^GUC, tRNA^Ala^CGC and tRNA^Arg^GCG transcripts. Thus, this result suggests that thermophilic archaea G^+^ formation system may possess broader substrate tRNA specificity than the mesophilic archaea system.

### The lysine transfer reaction mediated by ArcS prevents the reverse reaction in G^+^ formation in tRNA

**Figure 9:**
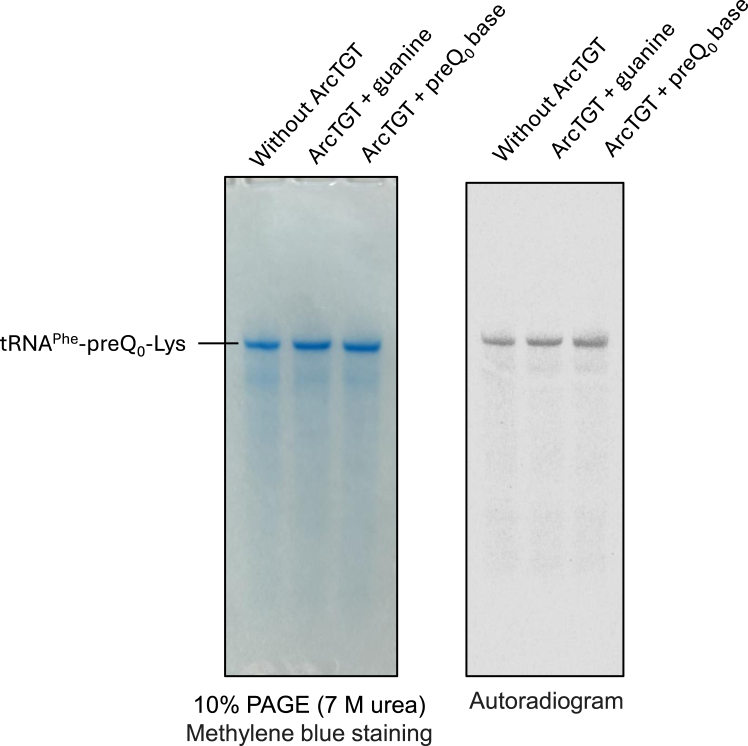
**ArcTGT does not exchange the preQ**_**0**_**-Lys in tRNA by guanine or preQ**_**0**_**base.** G15 in *S. cerevisiae* tRNA^Phe^ transcript was near-completely modified to preQ_0_-^14^C-Lys by the combination of ArcTGT and ArcS and RaSEA complex from *T. kodakarensis*. This tRNA transcript (0.1 A260 units each) was incubated in the buffer without proteins (*left*), with 0.5 μM ArcTGT and 200 μM guanine base (*middle*), and with 0.5 μM ArcTGT and 200 μM preQ_0_ base (*right*) at 60 °C for 2 h and then analyzed by 10% PAGE (7 M urea) (*left panel*). The RNAs were visualized by methylene blue staining. The band intensities in the autoradiogram (*right panel*) do not differ, demonstrating that ArcTGT does not exchange the preQ_0_-Lys in tRNA with guanine or preQ_0_ base.

ArcTGT can exchange the preQ_0_ base in tRNA with guanine base (25). This reverse reaction perturbs the formation of G^+^ in tRNA. If the intermediate (preQ_0_-Lys) prevents the reverse reaction, the lysine-transfer reaction mediated by ArcS would play a role in promoting the anterograde reaction (G^+^ formation). Therefore, we investigated whether ArcTGT can exchange the preQ_0_-Lys in tRNA with guanine or preQ_0_ base (Fig. 9). As a substrate, *S. cerevisiae* tRNA^Phe^-preQ_0_ was near-completely modified to tRNA^Phe^-preQ_0_-Lys with ^14^C-lysine. This tRNA^Phe^-preQ_0_-^14^C-Lys (8 μM) was incubated with buffer at 60 °C for 4 h (control; left lane in Fig. 9). Addition of 1.0 μM ArcTGT and 200 μM guanine base (middle lane) or 200 μM preQ_0_ base (right lane) did not change the intensities of tRNA^Phe^-preQ_0_-^14^C-Lys in the autoradiogram. This result shows that ArcTGT does not catalyze the reverse reaction from tRNA-preQ_0_-Lys to tRNA-guanine or tRNA-preQ_0_. Furthermore, ArcS does not catalyze the reverse reaction or lysine-exchange reaction of preQ_0_-Lys in tRNA. To demonstrate this, we performed one experiment (Fig. S4). The tRNA^Phe^-preQ_0_-^14^C-Lys (8 μM) was incubated with buffer at 60 °C for 4 h (control; left lane in Fig. S4). Addition of 0.11 μM ArcS and RaSEA complex (middle lane) and 0.11 μM ArcS and RaSEA complex and 200 μM lysine (right lane) did not change the band intensities of the autoradiogram. These experimental results are consistent with the result in Figure 4: 5′P-preQ_0_ nucleoside was near-completely converted to 5′P-preQ_0_-Lys, showing that the reverse reaction is not catalyzed by ArcS. Taking all experimental results together, we conclude that the formation of tRNA-preQ_0_-Lys by ArcS plays a role in preventing the reverse reaction in G^+^ biosynthesis.

## Discussion

In this study, we have focused on the substrate RNA recognition mechanism of ArcS. To our surprise, ArcS can act on preQ_0_ nucleoside and the presence of 5′-phosphate accelerates the lysine-transfer reaction. There are several tRNA modification enzymes, which have a broad substrate RNA specificity. For example, *S. cerevisiae* Trm4 (tRNA 5-methylcytosine methyltransferase) acts at multiple positions in tRNAs (46, 47, 48). Furthermore, the eukaryotic pseudouridine synthase, Pus7 acts at multiple positions in several RNA including tRNA (49, 50, 51). Moreover, archaeal NAT10 forms N4-acetylcytosine at multiple positions in several RNAs including tRNA (52). In addition, several tRNA modification enzymes can act on small RNA fragments such as microhelix RNAs (53, 54, 55, 56, 57, 58). However, these tRNA modification enzymes do not act on nucleoside or nucleotide. To our knowledge, ArcS is an exceptional tRNA modification enzyme that is able to act on nucleoside or nucleotide. In living cells, preQ_0_ nucleoside and preQ_0_ nucleotide are only produced by the degradation of tRNA-preQ_0_15, an intermediate of tRNA-G^+^15 formation. Because the Km value of ArcS for 5′P-preQ_0_ nucleoside is very large (433.4 μM), the lysine-transfer reaction to nucleoside or nucleotide by ArcS rarely occurs in cells.

ArcTGT from thermophilic archaea recognizes the ribose-phosphate backbone of the D-arm structure in tRNA (28). Although the loop and stem sizes of D-arm in archaeal tRNAs differ, several nucleosides (A13, pyrimidine17, G18, G19 and A21) including the modification site (G15) are highly conserved. Therefore, we considered whether the combination of ArcTGT and ArcS and RaSEA complex from *T. kodakarensis* might act on all tRNAs. In fact, preQ_0_ in all tRNA transcripts tested was modified to preQ_0_-Lys by ArcS *in vitro*. Furthermore, *H. volcanii* tRNAs, in which G15 is not modified to G^+^15 in living *H. volcanii* cells, were also modified by the ArcTGT and ArcS and RaSEA complex from *T. kodakarensis*. However, the velocities of formation of preQ_0_-Lys in tRNA differed between the tRNA transcripts (Fig. 6). For example, the velocity of formation of preQ_0_-Lys in the tRNA^Leu^CAG transcript was considerably slower compared to that in other tRNA transcripts. This phenomenon was caused by a slow lysine-transfer reaction to preQ_0_-tRNA^Leu^CAG transcript (Fig. 7). Thus, although ArcS can act on preQ_0_ nucleoside, the structure of substrate tRNA affects the lysine-transfer reaction mediated by ArcS. The presence of 5′-phosphate at the modification site (preQ_0_15) accelerates the lysine-transfer reaction, suggesting that ArcS captures this phosphate during the lysine-transfer reaction. In the complex between ArcTGT and tRNA, ArcTGT does not capture the 5′-phosphate at the modification site (G15) (30). These observations suggest a difference between the tRNA-binding modes of ArcS and ArcTGT although ArcS and ArcTGT share amino acid sequence homology (34). To clarify this in detail, structural studies of the complex of ArcS, RaSEA, and tRNA are necessary.

Until now, G^+^ has been found only in tRNAs (1). However, our experimental results suggest that the combination of ArcTGT and the ArcS and RaSEA complex may act on other RNAs except for tRNAs if the D-arm-like structure is contained in the RNA.

In general, the modification levels of tRNA in living cells depend on the quantitative balance between substrate tRNA and tRNA modification enzymes. For example, over-expression of Trm10 [tRNA methyltransferase for 1-methylguanosine at position 9 (m^1^G9); ref. (59)] in *S. cerevisiae* cells results in the m^1^G9 modification in tRNA species that are ordinarily unmodified *in vivo* (60). Similarly, although native tRNA^Phe^ from *E. coli* does not possess 2′-*O*-methylguanosine at position 18 (Gm18) (61), *E. coli* TrmH (tRNA methyltransferase for Gm18 modification; ref. (62, 63)) can methylate tRNA^Phe^ transcript very slowly under *in vitro* conditions (64). Therefore, some native tRNA species such as tRNA^Leu^CAG may possess unmodified G15 instead of G^+^15. This idea is in line with the observation that elongator tRNA^Met^ from *P. furiosus* possesses unmodified G15 (11), although *P. furiosus* is a hyper-thermophilic archaeon. In addition, it should be mentioned that a mesophilic archaeon, *H. volcanii*, may have a different G^+^15 modification system. Watanabe *et al.* purified native ArcTGT from *H. volcanii* cells (25). This ArcTGT efficiently catalyzes the ^14^C-guanine exchange reaction to *H. volcanii* tRNA^Lys^ transcript but not to *S. cerevisiae* and bovine tRNA mixtures (25). Therefore, ArcTGT from *H. volcanii* may select specific tRNAs as substrates.

In this study, we have clarified the substrate RNA specificity of ArcS. Furthermore, we have established a method for the preparation of tRNA-preQ_0_-Lys15. As shown in Figure 4*B*, preQ_0_-Lys was near-completely introduced into tRNA transcript. In fact, in this study, tRNA-preQ_0_-Lys15 was used for the experiments described in Figure 9 and Fig. S4. ArcTGT does not exchange the preQ_0_-Lys15 in tRNA with guanine or preQ_0_ base. Therefore, the formation of preQ_0_-Lys15 in tRNA prevents the reverse reaction in G^+^ biosynthesis pathway. In future studies, tRNA-preQ_0_-Lys15 will be useful for the analysis of the radical SAM reaction by RaSEA. Moreover, crystal structure studies may be possible using tRNA-preQ_0_-Lys15.

## Experimental procedures

### Materials

L-[^14^C(U)]-lysine (11,795.6 MBq/mmol) was purchased from PerkinElmer. Non-radioisotope-labeled lysine (L-lysine monohydrochloride) was obtained from Nacalai Tesque. [8-^14^C]-guanine hydrochloride (2123.8 MBq/mmol) was purchased from Moravek. DNA oligomers were obtained from Thermo Fisher Scientific. T7 RNA polymerase was purchased from Toyobo. All other chemical reagents were of analytical grade.

### Purification of *T. kodakarensis* ArcTGT

Purification of *T. kodakarensis* ArcTGT was described in our previous report (16).

### Purification of *T. kodakarensis* ArcS and RaSEA complex

Purification of *T. kodakarensis* ArcS and RaSEA complex was described in our previous report (19). In this study, the purification was performed under aerobic conditions.

### Preparation of tRNA transcripts

All tRNA transcripts were synthesized using T7 RNA polymerase as described previously (65). DNA oligomers used for the constructions of templates were designed using a Python tool, ROCKET, which was developed in our laboratory (45). The source code for ROCKET is available at Github (https://github.com/TEPPEI-MAT/ROCKET). The sequences of DNA oligomers are listed in Table S1. Two tRNA^Gln^ and initiator tRNA^Met^ from *T. kodakarensis* do not possess G nucleotide at position 1. Therefore, these tRNAs were prepared as precursor forms, which have an attached 5′-leader sequence, which was removed by RNase P digestion as described in a previous report (66). In Figure 6, tRNA^Gln^CUG RNase P, tRNA^Gln^UUG RNase P, and tRNA^Met^CAU RNase P were prepared by the method using RNase P. The purification procedure of RNase P is described in the reference (66). Transfer RNA transcripts were purified by 10% PAGE (7 M urea).

### Base, nucleoside, and nucleotide

The guanine base was purchased from Sigma. preQ_0_ base was chemically synthesized according to the reference (67). preQ_0_ nucleoside 5′-phosphate was synthesized as a triethylammonium salt by 5′-phosphorylation (68) of preQ_0_ nucleoside (69). ^1^H NMR (400 MHz, D_2_O) δ 7.85 (s, 1H), 5.98 (d, *J* = 6.8 Hz, 1H), 4.57 (dd, *J* = 6.8, 5.2 Hz, 1H), 4.37 (dd, *J* = 5.2, 2.9 Hz, 1H), 4.22 (q, *J* = 2.9 Hz, 1H), 3.93–3.84 (m, 2H), 3.07 (q, *J* = 7.3 Hz, 12H), 1.18 (t, *J* = 7.3 Hz, 18H). ^31^P NMR (161.7 MHz, D_2_O) δ 4.6 (s, 1P). HRMS (ESI-TOF) *m*/*z*: [M − H]^−^ calculated for C_12_H_13_N_5_O_8_P^–^ 386.0507; found 386.0513. The synthesis of 5′P-preQ_0_-Lys nucleoside was described in our previous report (19). Standard markers of preQ_0_-Lys nucleoside, 5′P-preQ_0_ nucleoside, and 3′P-preQ_0_ nucleoside in Figure 4 were prepared by nuclease digestions of tRNA^Phe^-preQ_0_-Lys. Procedures for preparations of these markers are described in the latter section “Lysine-transfer reaction to preQ0 base, preQ0 nucleoside and preQ0 nucleotides”.

### Preparation of *S. cerevisiae* tRNA^Phe^-preQ_0_

50.0 A260 units of *S. cerevisiae* tRNA^Phe^ transcript, 1 μM ArcTGT, and 200 μM preQ_0_ base were incubated in 6.25 ml of buffer A [50 mM Tris-HCl (pH 7.6), 5 mM MgCl_2_, 6 mM 2-mercaptoethanol, 50 mM KCl] at 60 °C for 2 h. After the reaction, RNA was treated with phenol-chloroform and recovered by ethanol precipitation.

### Measurements of kinetic parameters of ArcS for lysine, tRNA^Phe^-preQ_0,_ and RNA fragments

For measurement of kinetic parameters for lysine, 0.23 μM ArcS and RaSEA complex, 204 μM tRNA^Phe^-preQ_0_, and various concentrations of ^14^C-labeled lysine were incubated in 10 μl of buffer A at 60 °C for 5 min and spotted onto a Whatman 3 MM filter. Incorporations of ^14^C-lysine were monitored by conventional filter assay. In brief, the filters were washed in 50 ml 5% trichloroacetic acid solution five times. The filters were dipped into 50 ml 99.5% ethanol to remove water and then dried. The ^14^C-lysine incorporation was measured by a liquid scintillation counter. For measurement of kinetic parameters for tRNA-preQ_0_ or RNA fragment, 0.11 μM ArcS and RaSEA complex, 200 μM ^14^C-lysine, and various concentrations of tRNA^Phe^-preQ_0_ (or RNA fragment) were incubated in 10 μl of buffer A at 60 °C for 5 min. The aliquot was spotted onto a Whatman 3 MM filter and then incorporations of ^14^C-lysine were monitored by the filter assay described above. The data were obtained from three independent experiments. Km and Kcat values were calculated using a Python program developed in our laboratory. This program fits the data to the Michaelis-Menten equation. Error bars show the standard deviations. The source code of the program is described in Supporting Information.

### Cleavage of tRNA^Phe^-preQ_0_ by DNAzymes

In this study, two 8-17 DNAzymes were designed. The sequences are as follows: 8-17 DNAzyme for cleavage between A21 and G22, 5′- CTT CAG TCT GGC GCT TCC GAG CCG GAC GAC CCA ACT GAG CTA AA -3’; 8-17 DNAzyme for cleavage between A64 and G65, 5′- TGG TGC GAA TTT CCG AGC CGG ACG AGT GGA TCG AAC ACA GGA -3’. 0.2 A260 units tRNA^Phe^-preQ_0_ (final concentration 17 μM), and 25 μM 8-17 DNAzyme was mixed in 20 μl of DNAzyme buffer [20 mM Tris-HCl (pH 7.6), 50 mM MgCl_2_, 50 mM NaCl]. The cleavage was performed in a Gene Atlas Thermal Cycler model 482 (ASTEC). The sample was heated at 80 °C for 1 min, gradually cooled to 37 °C over 430 s, and incubated at 37 °C for 30 min. This cycle was repeated ten times and then incubated at 80 °C for 2 min. The RNA fragments were purified by 10% PAGE (7 M urea).

### Lysine-transfer reaction to RNA fragments containing preQ_0_

0.11 μM ArcS and RaSEA complex, 8.5 μM RNA fragment, and 100 μM ^14^C-lysine were incubated in 20 μl of buffer A at 60 °C for 10 min. RNA was treated with phenol-chloroform and recovered by ethanol precipitation. 0.03 A260 units each of RNA was analyzed by 10% PAGE (7 M urea). The gel was stained with 0.2% methylene blue and then dried. The incorporation of ^14^C-lysine into RNA was monitored using a Typhoon FLA 7000 system (Cytiva). The kinetic parameters for RNA fragments containing preQ_0_ were described in the “Measurements of kinetic parameters of ArcS for lysine, tRNAPhe-preQ0, and RNA fragments” section.

### Lysine-transfer reaction to preQ_0_ base, preQ_0_ nucleoside and preQ_0_ nucleotides

Base, nucleoside, and nucleotide analyses were performed with a Hitachi L-2200 HPLC system (Hitachi) equipped with a Nucleosil 7C18 column (4.6 × 250 mm; ChemcoPlus Scientific). The solvent system was previously reported (70). The proteins in the samples were removed using a Durapore Centrifugal Filter Device (PVDF, 0.22 mm; Merck Millipore) and then the flow-through fraction was analyzed. For preQ_0_ base analysis (Fig. 4*A*), 0.23 μM ArcS and RaSEA complex, 17 μM preQ_0_ base and 200 μM lysine were incubated in 20 μl of buffer A at 60 °C for 2 h. For preQ_0_ nucleoside analysis, *S. cerevisiae* tRNA^Phe^-preQ_0_ (0.2 A260 units) was digested with 2.5 units nuclease P1 (Fuji Film-Wako) in 18 μl 50 mM sodium acetate (pH 5.3) at 37 °C for 2 h. After the nuclease P1 digestion, 0.5 units bacterial alkaline phosphatase and 1 μl 1 M Tris-HCl (pH 8.0) were added into the sample and then incubated at 37 °C overnight. This sample is used as “Before reaction” in Figure 4*B*. In the case of “Standard” in Figure 4*B*, 0.23 μM ArcS and RaSEA complex, 0.2 A260 units *S. cerevisiae* tRNA^Phe^-preQ_0_ and 200 μM lysine were incubated in 20 μl of buffer A at 60 °C for 2 h. The RNA was treated with phenol-chloroform and recovered by ethanol precipitation. The RNA was digested with nuclease P1 and alkaline phosphatase as described above. In the case of “After reaction”, 0.2 A260 units *S. cerevisiae* tRNA^Phe^-preQ_0_ was digested to nucleosides with nuclease P1 and alkaline phosphatase as described above. 0.23 μM ArcS and RaSEA complex and 10 x buffer A were added into the sample and then incubated at 60 °C for 2 h. For 5′P-preQ_0_ nucleoside analysis (Fig. 4*C*), *S. cerevisiae* tRNA^Phe^-preQ_0_ (0.2 A260 units) was digested with 2.5 units nuclease P1 in 18 μl 50 mM sodium acetate (pH 5.3) at 37 °C for 2 h. This sample was used for “Before reaction” in Figure 4*C*. In the cases of “Standard” and “Before reaction” in Figure 4*C*, the samples were not treated with bacterial alkaline phosphatase. For 3′P-preQ_0_ nucleoside analysis (Fig. 4*D*), *S. cerevisiae* tRNA^Phe^-preQ_0_ (0.2 A260 units) was digested with 5 units RNase T2 (Sankyo) in 20 μl ammonium acetate (pH 5.0). The 3′phosphate-nucleosides from tRNA were analyzed using the same method as described above.

### Spectroscopic analysis of lysine-transfer reaction

UV absorption spectra of 50 μM 5′P-preQ_0_ and 50 μM 5′P-preQ_0_-Lys in buffer B [50 mM Tris-HCl (pH 7.6), 5 mM MgCl_2_, 50 mM KCl] were recorded at 60 °C using an Ultrospec 6300 pro photometer (GE Healthcare). The initial velocity of the lysine-transfer reaction to 5′P-preQ_0_ in Figure 5*B* was determined as follows. 100 μM 5′P-preQ_0_ and 200 μM lysine in 400 μl buffer B were prewarmed at 60 °C and the reaction was started by the addition of ArcS and RaSEA complex (final concentration is 0.11 μM). The kinetic parameters were calculated from the velocities of lysine-transfer to various concentrations of 5′P-preQ_0_.

### Measurements of ^14^C-lysine-incorporations into tRNA transcripts

1.0 μM ArcTGT, 0.23 μM ArcS and RaSEA complex, 0.1 A260 units tRNA transcript, 500 μM preQ_0,_ and 100 μM lysine were incubated in 20 μl buffer A at 60 °C for 5 min. To remove K^+^ ions, the buffer A in the samples was exchanged with water using an Amicon Ultra Centrifugal Filter device (Millipore, 10,000 MWCO, code number UFC501024). RNAs were treated with phenol-chloroform and recovered by ethanol precipitation. 0.01 A260 units RNAs were analyzed by 10% PAGE (7 M urea). The gels were stained with 0.2% methylene blue and then dried. Autoradiograms of the gels were obtained using a Typhoon FLA 7000 system (Cytiva).

### Analysis of base exchange reaction by ArcTGT

0.30 A260 units *S. cerevisiae* tRNA^Phe^-preQ_0_, 0.23 μM ArcS and RaSEA complex and 300 μM ^14^C-lysine were incubated in 30 μl buffer A at 60 °C for 2 h. RNA was treated with phenol-chloroform and then recovered by ethanol precipitation. The resultant tRNA^Phe^-preQ_0_-^14^C-Lys was dissolved in 20 μl water. 0.10 A260 units tRNA^Phe^-preQ_0_-^14^C-Lys, 0.5 μM ArcTGT and 200 μM guanine base (or preQ_0_ base) were incubated in 20 μl buffer A at 60 °C for 2 h. 0.03 A260 units RNAs were analyzed by 10% PAGE (7 M urea). The gels were stained with 0.2% methylene blue and then dried. Autoradiogram of the gel was obtained using a Typhoon FLA 7000 system (Cytiva). For the experiment in Figure 7*B* left, 0.10 A260 units *T. kodakarensis* tRNA^Leu^CAG (or tRNA^Trp^), 85 μM ^14^C-guanine, and 0.75 μM ArcTGT were incubated in 20 μl buffer A at 60 °C for 0, 2, 5, 10, and 20 min. The incorporation of ^14^C-guanine was monitored by filter assay as described in the previous section. For the experiment in Figure 7*B* right, 0.10 A260units *T. kodakarensis* tRNA^Leu^CAG-preQ_0_ (or tRNA^Trp^-preQ_0_), 300 μM ^14^C-lysine and 0.11 μM ArcS and RaSEA complex were incubated in 20 μl buffer A at 60 °C for 0, 1, 2, 5, 10, 15, and 20 min. The incorporation of ^14^C-lysine was monitored by filter assay as described in the previous section.

## Data availability

All data used in this study are available from the corresponding author (H. H.) upon request.

## Supporting information

This article contains supporting information.

## Conflict of interest

The authors declare that they have no conflicts of interest with the contents of this article.
