# Supplementary material for: ArcS from Thermococcus kodakarensis transfers L-lysine to preQ0 nucleoside derivatives as minimum substrate RNAs
Source: J Biol Chem. 2024 Jun 27;300(8):107505. doi: 10.1016/j.jbc.2024.107505 (PMC11298593; doi:10.1016/j.jbc.2024.107505)
Supplement: Supporting Information [file mmc2.pdf]

Supporting information

ArcS from *Thermococcus kodakarensis* transfers L-lysine to preQ$_0$ nucleoside derivatives as minimum substrate RNAs.

Shu Fujita[1], Yuzuru Sugio[1], Takuya Kawamura[1*], Ryota Yamagami[1], Natsuhisa Oka[2, 3, 4], Akira Hirata[5], Takashi Yokogawa[2, 4, 6], and Hiroyuki Hori[1]**

1. Department of Materials Science and Biotechnology, Graduate School of Science and Engineering, Ehime University, 3 Bunkyo-cho, Matsuyama, Ehime 790-8577, Japan
2. Department of Chemistry and Biomolecular Science, Faculty of Engineering, Gifu University, 1-1 Yanagido, Gifu, Gifu 501-1193, Japan
3. Institute for Glyco-core Research (iGCORE), Gifu University, 1-1 Yanagido, Gifu, Gifu 501-1193, Japan
4. Center for One Medicine Innovative Translational Research (COMIT), Gifu University, 1-1 Yanagido, Gifu, Gifu 501-1193, Japan
5. Department of Natural Science, Graduate School of Technology, Industrial and Social Science, Tokushima University, 2-1 Minamijosanjimacho, Tokushima, Tokushima 770-8506, Japan
6. United Graduate School of Drug Discovery and Medical Information Sciences, Gifu University, 1-1 Yanagido, Gifu, Gifu 501-1193, Japan

*Present address:
Computational Medicine Center, Sidney Kimmel Medical College, Thomas Jefferson University, Philadelphia, Pennsylvania, USA

** To whom correspondence should be addressed:
Hiroyuki Hori
Department of Materials Science and Biotechnology, Graduate School of Science and Engineering, Ehime University, 3 Bunkyo-cho, Matsuyama, Ehime 790-8577, Japan.
Phone: +81-89-927-8548
E-mail: hori.hiroyuki.my@ehime-u.ac.jp

Experimental Procedures

Interaction analysis between ArcS, RaSEA and ArcTGT by pull-down assay for SFig. 1

The plasmid pETY-MA0121-MA4419, the IPTG-inducible vector for the split-type MaArcTGT without His-tag, was constructed from pETY-MA0121-MA4419-His_6 (19) by introducing a stop codon into the upstream of the His-tag sequence. Then, *E. coli* strain ER2566 harboring pETY-MA0121-MA4419 and pHara-MA4513-SD-MA4632-His_6 (19), the coexpression vector which is compatible with pETY-MA0121-MA4419, was grown in 200 ml of LB with 50 µg/ml ampicillin and 34 µg/ml chloramphenicol at 37°C until the cell density reached OD_{600} = 0.8. The culture was then divided into 100 ml each. One for preparing the cell extract without induction was further cultivated at 37°C for 4 h and then kept static at 4°C overnight. To induce the production of MaArcS and MaRaSEA in the remaining culture, arabinose was added to a final concentration of 0.2% (w/v) and, at the same time, the cells were supplemented with $FeCl_3$, $Fe(NH_4)_2(SO_4)_2$ and cysteine to final concentrations of 50, 50 and 400 µM, respectively. After further cultivation at 37°C for 1 h, IPTG was added to a final concentration of 0.5 mM to induce the production of MaArcTGT and the cells were further cultivated at 37°C for 3 h and then kept static at 4°C overnight. The harvested cells were suspended in 10 ml of lysis buffer (20 mM Tris–HCl [pH 7.6], 1 mM $MgCl_2$, 0.2 M KCl, 6 mM 2-mercaptoethanol and 5% [v/v] glycerol) and disrupted by sonication. After removing cell debris by centrifugation, the cell extract without induction was stored at –80°C until analysis. The cell extract with induction was

loaded onto a Ni-NTA agarose column (0.4 ml; FUJIFILM Wako) pre-equilibrated with lysis buffer. The column was washed with 10 ml of wash buffer (20 mM Tris–HCl [pH 7.6], 1 mM $MgCl_2$, 0.2 M KCl, 6 mM 2-mercaptoethanol, 5% [v/v] glycerol and 10 mM imidazole) twice. Then, proteins were eluted with 2 ml of elution buffer (20 mM Tris–HCl [pH 7.6], 1 mM $MgCl_2$, 0.2 M KCl, 6 mM 2-mercaptoethanol, 5% [v/v] glycerol and 250 mM imidazole) twice. An appropriate aliquot (2.5-10 µl) of each fraction was analyzed by 10% SDS-PAGE and the gel was stained with CBB.

Figure Legends

SFig. 1. ArcS forms a complex with RaSEA but bot with ArcTGT. 6 x His ArcS was co-expressed with RaSEA and ArcTGT in *E. coli* cells. Lane 1, cell extract before the induction; lane 2, cell extract after the induction. The cell extract was loaded onto a Ni-NTA agarose column and bound proteins were eluted by elution buffer, which contained 250 mM imidazole. Lane 3, flow-through fraction; lane 4, wash fraction 1; lane 5 wash fraction 2; lane 6, elution fraction 1; lane 7, elution fraction 2. RaSEA was co-purified with 6 x His ArcS(lane 6). In contrast, ArcTGT was eluted in the flow-through and wash fractions.

SFig. 2. 15% SDS-PAGE analyses of ArcTGT and ArcS and RaSEA complex. A, 5 μg of *T. kodakarensis* ArcTGT was analyzed by 15% SDS-PAGE. **B**, 10 μg of *T. kodakarensis* ArcS and RaSEA complex was analyzed by 15% SDS-PAGE. The gels were stained with Coomassie Brilliant Blue.

SFig. 3. Preparation of $tRNA^{Gln}$UUG and $tRNA^{Gln}$CUG transcripts. Because $tRNA^{Gln}$UUG and $tRNA^{Gln}$CUG transcripts possess A at the 5'-end, synthesis by T7 RNA polymerase is difficult. Therefore, these tRNA transcripts were prepared as precursor forms (**A**) and then digested with RNaseP. Arrows show the cleavage sites of RNaseP. The resultant transcripts were purified by 10% PAGE (7 M urea). In Fig. 6, these tRNA transcripts are labeled as Gln UUG RNase P and Gln CUG RNase P. In Fig. 6, Gln UUG G-C and Gln CUG G-C possess the artificial G1-C72 base pair: the cloverleaf structures are shown in panel (**B**). The replaced base pairs are enclosed in red squares.

SFig. 4. ArcS does not catalyze the reverse reaction and lysine-exchange reaction. G15 in *S. cerevisiae* $tRNA^{Phe}$ transcript was near-completely modified to $preQ_0$-^{14}C-Lys by the combination of ArcTGT and ArcS and RaSEA complex from *T. kodakarensis*. This tRNA transcript (0.1 A260 units each) was incubated in the buffer without proteins (left), with 1.0 μM ArcS (middle), and with 1.0 μM ArcS and 200 μM non-radioisotope labeled lysine (right) at 60°C for 2 h and then analyzed by 10% PAGE (7 M urea) (left panel). The RNAs were visualized by methylene blue staining. The band intensities in the autoradiogram (right panel) do not differ, demonstrating that ArcS does not catalyze the reverse reaction from $preQ_0$-Lys to $preQ_0$ in tRNA and the lysine-exchange reaction.

Supporting Table 1

The sequences of tRNA transcripts and DNA oligomers are listed in an excel file.

The source code of Python software for kinetic study

```
#import module#
import pandas as pd
import numpy as np
import matplotlib.pyplot as plt
from scipy.optimize import curve_fit


#100,000 dpm / (2.22 x 1012 dpm / Ci) = 4.5 x 10 -8 Ci
#4.5×10-8 Ci x (1mmol/83.2Ci) =5.4×10-10mmol=0.54pmol

#define finction#
def dpm_to_picomol(dataframe):
    return dataframe.loc["dpm"]*4/(2.22*10**9)/318.8*10**9

def MM(x,Vmax,km):
    return  Vmax*x/(x+km)

def graph_plot(max, Vmax, km):
  limit = max + max/10
  x = []
  y = []
  lt = range(0,100 + 1)
  for j in lt:
    i = max/100 * j
    x.append(i)
    y.append(MM(i,Vmax,km))
  return x, y
ue=#amount of enzyme
time=#reaction time min
df = pd.read_csv("21ntkine.csv")#read csvfile
df["mol"] = df.apply(dpm_to_picomol,axis = 1)/time
df1 = df.groupby("conc",as_index = False,sort = False).describe()["mol"]
df2 = df.query("replicates == 'rep1'")
df1["conc"] = df2["conc"]
df3,cov = curve_fit(MM,df["conc"],df["mol"],p0=[0, 0])
xs, ys = graph_plot(df1["conc"].max(),df3[0],df3[1])
df4 = pd.DataFrame({'conc': xs, 'mol': ys})
Vmax = df3[0]
Km = df3[1]
Kcat=df3[0]/ue
fig,ax = plt.subplots()
x = df4["conc"]
y = df4["mol"]
```

```
yerr = df1["std"]
plt.rcParams["font.size"] = 20
ax.plot(x,y,color = "#1e1210")
x1 = df1["conc"]
y1 = df1["mean"]
x2 = df["conc"]
y2 = df["dpm"]*4/(2.22*10**9)/318.8*10**9/time
ax.errorbar(x1,y1,yerr = yerr,ecolor = "#1e1210",capsize = 7,fmt = "go",markersize=0,capthick = 2,elinewidth = 2)
ax.scatter(x2,y2,color = "turquoise",edgecolor = "black",s = 50)
V = "Vmax = "+str(round(Vmax,1))+" [pmol/min]"
K = "Km = "+str(round(Km,1))+" [μM]"
C='Kcat ='+str(round(Kcat,1))+"[/min]"
print(K)
print(V)
print(C)
plt.savefig("",transparent=True,dpi = 600,bbox_inches='tight')# filename
```

SFig. 1

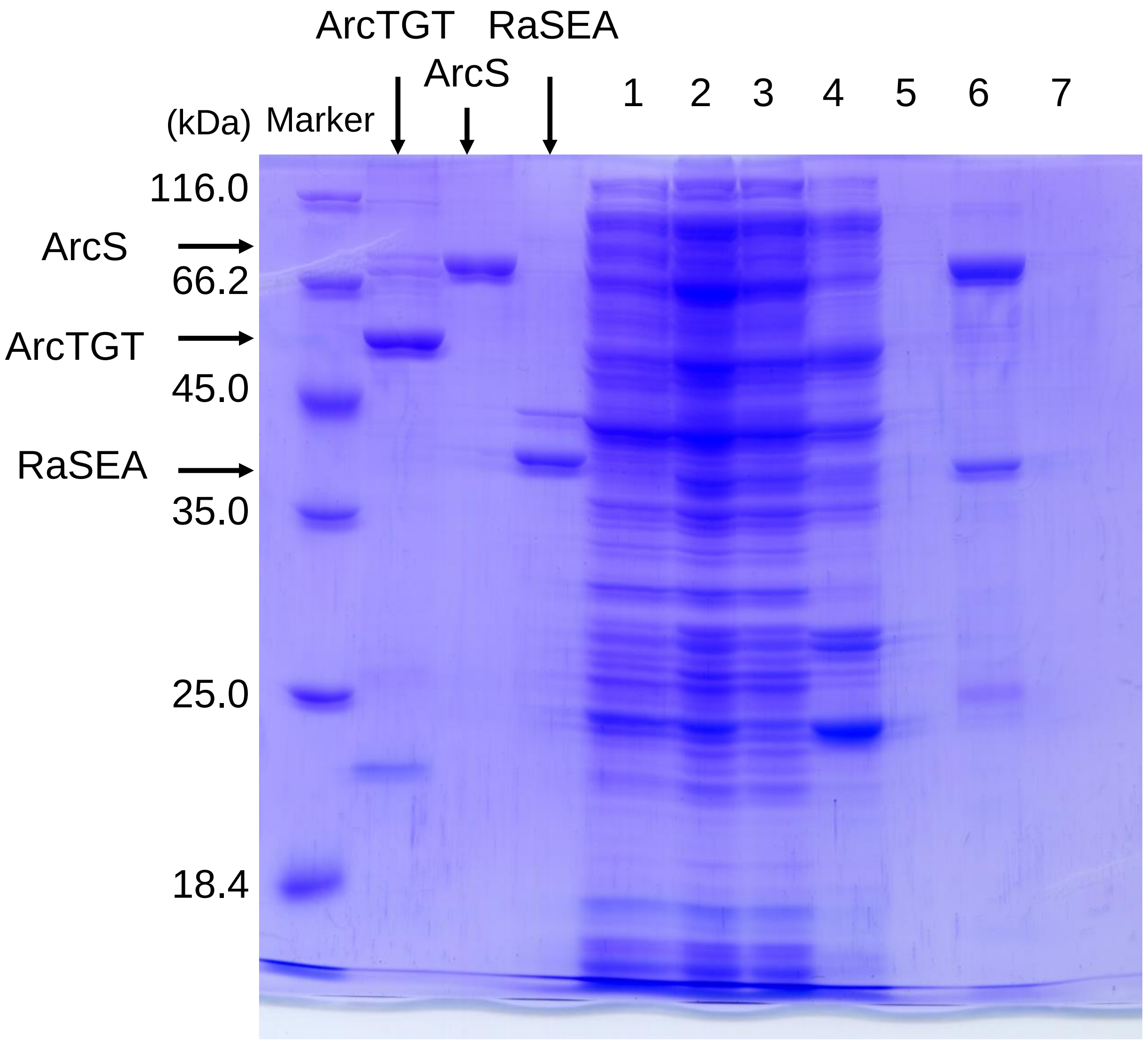

10% SDS-PAGE/ Coomassie Brilliant Blue staining

SFig. 2

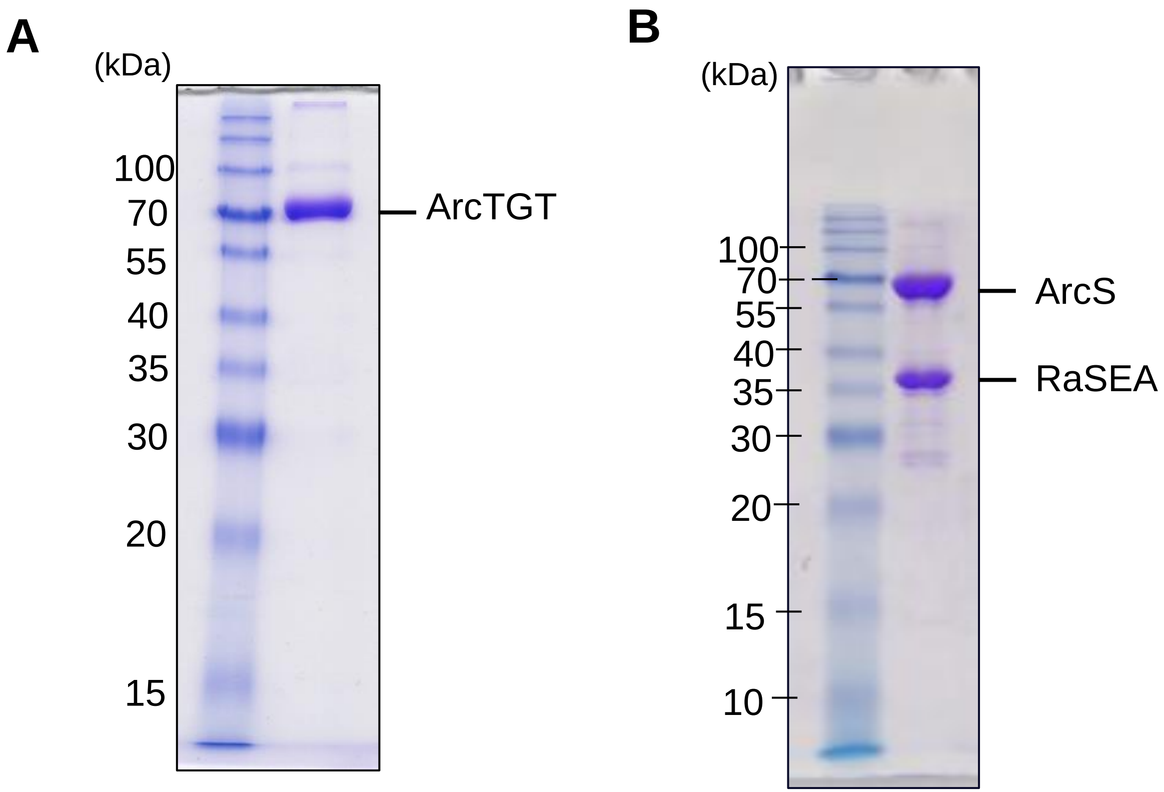

15% SDS-PAGE
Coomassie brilliant blue staining

15% SDS-PAGE
Coomassie brilliant blue staining

SFig.3

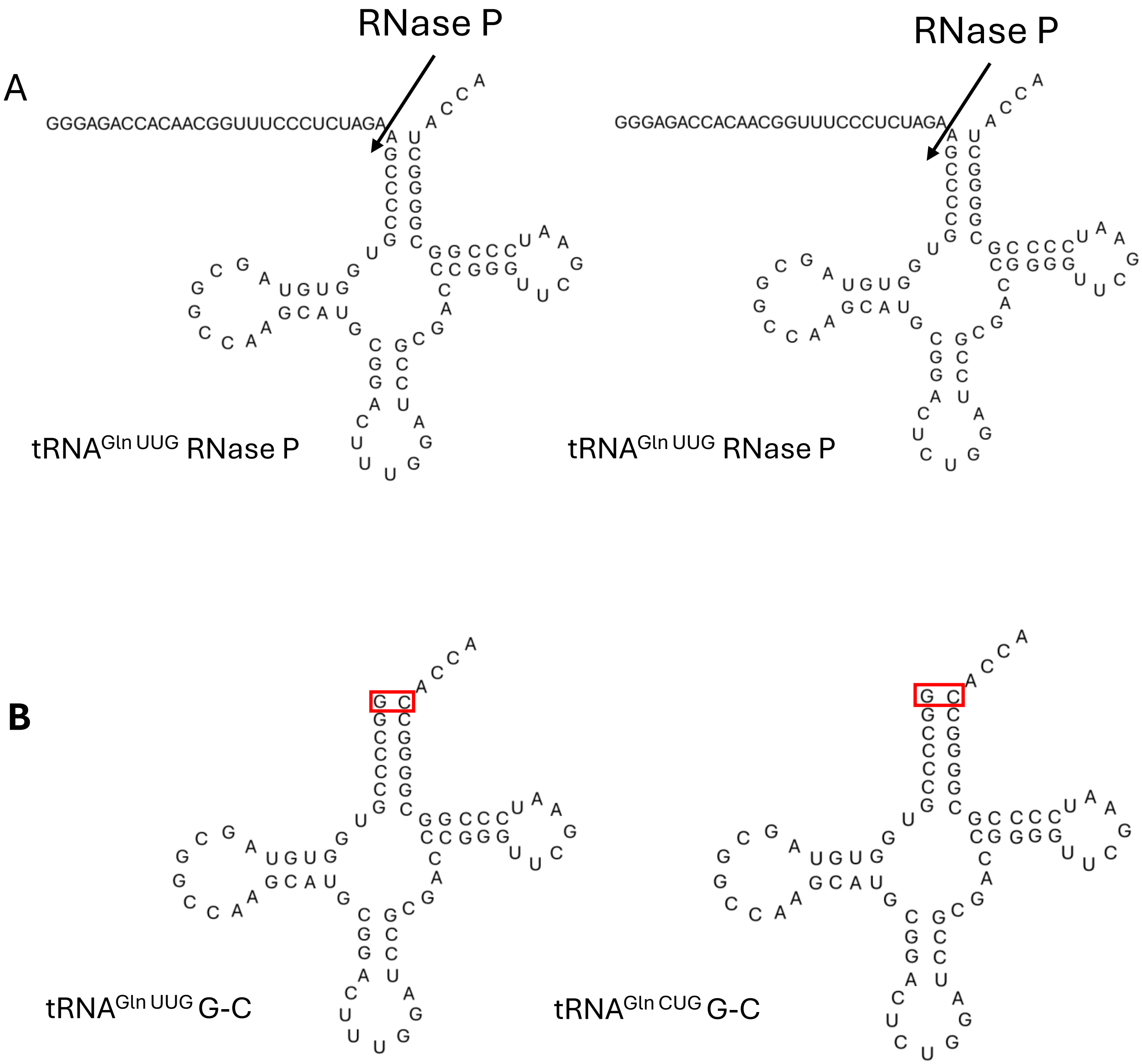
A
RNase P
GGGAGACCACAACGGUUUCCCUCUAGA
tRNA$^{Gln\ UUG}$ RNase P
RNase P
GGGAGACCACAACGGUUUCCCUCUAGA
tRNA$^{Gln\ UUG}$ RNase P
B
tRNA$^{Gln\ UUG}$ G-C
tRNA$^{Gln\ CUG}$ G-C

SFig. 4

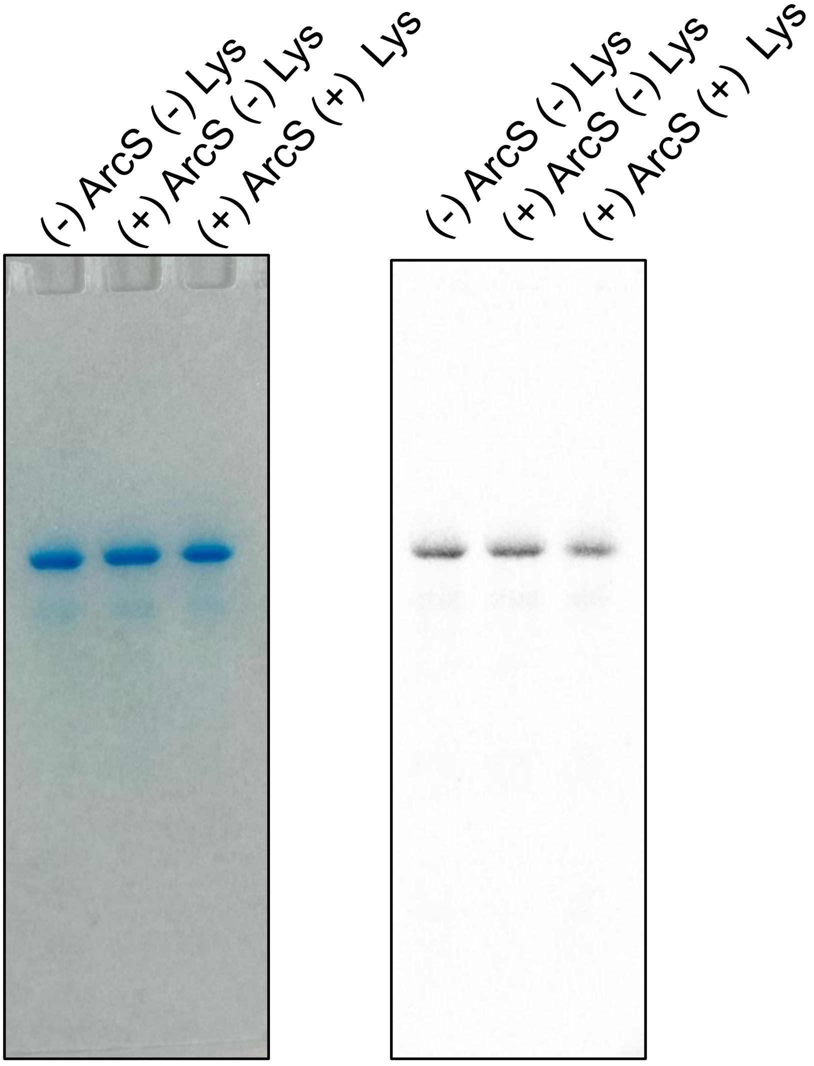

10% PAGE (7 M urea)
Methylene blue staining

Autoradiogram
